# Perspectives on non-target site mechanisms of herbicide resistance in weedy plant species using evolutionary physiology

**DOI:** 10.1093/aobpla/plx035

**Published:** 2017-07-29

**Authors:** Hossein Ghanizadeh, Kerry C Harrington

**Affiliations:** 1 Institute of Agriculture and Environment, Massey University, PB 11-222, Palmerston North 4442, New Zealand

**Keywords:** Evolution, herbicide resistance, mechanism of resistance, physiology, weeds

## Abstract

Evolutionary physiology merges the disciplines of evolution and physiology, and it is a research approach that has not received much attention for studying the development of herbicide resistance. This paper makes a case for using evolutionary physiology more frequently when studying herbicide resistance, and illustrates this using three areas where more work would be useful: (i) the interaction among major and minor alleles over many generations during the evolution of physiological responses that lead to specific mechanisms of resistance; (ii) the role of epigenetic factors, especially at an early stage of evolution, on the physiological modifications that result in phenotypes that become insensitive to herbicides; and (iii) the interaction between fitness and physiological performance over time, with emphasis on understanding mechanisms that improve the fitness of herbicide-resistant phenotypes during selection.

## Teaser

Research into evolutionary physiology involves principles from both the disciplines of evolution and physiology. However investigations into development of resistance to herbicides have often not given much consideration of evolutionary physiology. This paper suggests that a better understanding of herbicide resistance in weeds should be possible by investigating the evolutionary physiology involved.

## Introduction

Application of herbicides has been a crucial part of the global weed management strategy (DiTomaso 2002). While herbicides have greatly improved agricultural production, their usefulness is now being compromised by the evolution of resistance in many major weedy plant species (Heap 2014). New cases of herbicide resistance are being reported every year globally (Heap 2017). The evolution of herbicide-resistant weed populations is the adaptive response of weed populations to the selection pressures exerted by persistent applications of herbicides with the same mode of action (Neve 2007). Herbicide resistance is a good example of the adaptability of plant species, making it an interesting topic for evolutionary biologists (Neve *et al.* 2009).

Studies have been conducted worldwide on the molecular and physiological mechanisms governing herbicide resistance to weedy plant species, which help with the development of more effective strategies to prevent resistance from occurring and also to control resistant weed populations (Powles and Yu 2010). Mechanisms of resistance to herbicides are categorized as being either ‘target site’ or ‘non-target site’ in nature. Target site mechanisms of resistance may involve structural modifications of a target enzyme so herbicides are no longer able to fit exactly to the site of action (Devine and Shukla 2000), or gene amplification/overexpression of the target site, in which the target protein can be produced in large quantities by the plant (Gaines *et al.* 2010). With non-target site mechanisms, the number of herbicide molecules reaching the target site is reduced, either due to detoxification of herbicides to non-toxic metabolites (enhanced metabolism), or sequestration to other parts of plant cells (e.g. within vacuoles) (Ghanizadeh and Harrington 2017).

The non-target site mechanisms of resistance to herbicides tend to be more complicated than target site mechanisms and are often part of plant stress responses which evolve through time (Délye 2013). Previous studies have focused more on the comparative physiology (e.g. metabolism rate) and physiological ecology (e.g. thermoregulatory performance) of the non-target mechanism of herbicide resistance (Dayan *et al.* 2014; Sammons and Gaines 2014). The historical patterns and process of physiological evolution of these non-target site mechanisms of herbicide resistance are poorly understood.

Evolutionary physiology is a combined approach of physiology and evolutionary biology to study how and why the functioning of an organism evolves (Feder *et al.* 2000). Evolutionary physiologists use functional approaches to understand how the physiological characteristics of an organism adapt to a wide range of biotic and abiotic environments over a period of time (Garland and Carter 1994). Despite considerable research on herbicide-resistant weed biotypes, many aspects of the physiological adaptation of these biotypes to herbicides are poorly understood. For example, there are a number of studies on the evolutionary biology of herbicide-resistant biotypes (e.g. Busi *et al.* 2013). However, there is a poor understanding of the physiological processes encoded by the genes that have been selected by persistent herbicide use, and the impacts of these processes on the evolution of herbicide resistance.

To have a better understanding of the physiological functions involved in mechanisms of herbicide resistance, more needs to be known about their origin and development (Feder *et al.* 2000). Although evolutionary physiology has been successfully used in human, animal and plant science (Natochin and Chernigovskaya 1997; Feder *et al.* 2000), this biological approach to study evolution of resistance to herbicides has not received much attention. The objective of this paper is to outline some perspectives regarding the evolutionary physiology of non-target site mechanisms of herbicide resistance and to suggest directions for future studies.

## Perspectives on Non-target Site Mechanisms of Herbicide Resistance

### Interactions among alleles during evolution

Following exposure of plant populations to adverse environmental conditions, they often evolve complicated stress-response systems over many generations which enable them to adjust to the environment that they inhabit (Cramer *et al.* 2011). These systems can undergo several modifications as populations face new threats from the environment (Yoshida 2005). Individuals within the population with appropriate heritable alleles survive the environmental stress and can contribute to subsequent generations.

Studies using populations which were initially susceptible to a herbicide have shown that weed populations are capable of evolving resistance to herbicides after 3–4 generations of recurrent applications of sublethal doses of a herbicide (Busi *et al.* 2013). The selection imposed by herbicides could lead to an accumulation of several alleles that cause some physiological modifications within each generation (Yoshida 2005), resulting in resistance to higher concentrations of the herbicides (Délye 2013). But how do these alleles modify the physiological function of individuals within each generation during the evolution? Also, how do these alleles interact with each other within each generation, and how does this interaction lead to a specific mechanism of resistance?

According to the ‘allele stacking theory’ (Délye 2013), over several generations, progeny plants of the individual plants that survived the application of herbicides accumulate different parental alleles that allowed the progeny plants to become less sensitive to the applied herbicides compared with their parental plants. The accumulation of several alleles in individual plants during the recurrent selection pressure from herbicides could lead to more genetic and physiological variations, thus modifying the physiology of species towards adaptation to herbicides across generations. For instance, Yu *et al.* (2013) found that recurrent selection of a *Lolium rigidum* population with sublethal doses of diclofop-methyl resulted in the evolution of enhanced diclofop-methyl metabolism. This enhanced metabolism was due to increased activity of cytochrome P450 enzymes (Gaines *et al.* 2014). Studies investigating the pattern of inheritance of cytochrome P450 metabolism have shown that this mechanism is governed by two additive genes (Busi *et al.* 2011). These two genes would accumulate during the selection process, according to the ‘allele stacking’ theory. However, it is not known how these two genes, which have accumulated over several generations, then interacted within each generation to eventually change the physiology of plants sufficiently to increase the cytochrome P450 metabolism enough to cause herbicide resistance. This raises several questions. How did each of these two genes contribute to herbicide metabolism when they were not present in an individual plant simultaneously? Which one of these two genes contributed the most at the early stage of developing resistance to herbicides?

Many studies have shown that minor genes can play a role in the mechanisms of resistance to herbicides (Lorraine-Colwill *et al.* 2001; Busi and Powles 2009; Busi *et al.* 2013; Ghanizadeh *et al.* 2016). It would be interesting to know more about the contribution of these minor genes during early stages of the evolution of herbicide resistance, and the nature of the interaction between these genes that allowed development of the resistance trait.

### The impact of epigenetic factors

The epigenetic landscape of an organism can be altered by environmental factors (Richards *et al.* 2010). Well-known epigenetic regulatory mechanisms include DNA methylation, such as the addition of a methyl group to cytosine nucleotides in DNA (Goll and Bestor 2005), also histone modification, and RNA-mediated modifications (Rapp and Wendel 2005). Although the impact of epigenetic processes in gene regulation due to stress is well documented (Boyko and Kovalchuk 2008), and the role of epigenetic mechanisms in insecticide resistance has been noted (Bass and Field 2011), the role of epigenetic processes in the evolution of herbicide-resistant weedy plants is still unknown.

Epigenetic mechanisms can change the patterns of gene expression as a result of the stress induced by biotic and abiotic agents. In the case of herbicides (as abiotic stress agents), it would be interesting to investigate how epigenetic mechanisms influence the pattern of gene expression in individual plants from populations subjected to gradual increases of herbicide doses (recurrent selection). It would also be interesting to know how epigenetic mechanisms might control the developmental processes underlying physiological responses in herbicide-resistant individuals. For instance, epigenetic factors can influence the level of gene expression in a heritable fashion across the generations involved with the evolution of herbicide resistance, affecting the activity of enzymes involved in the resistance mechanism (Lee *et al.* 2010).

Studies of non-target site mechanisms of herbicide resistance using RNA-sequencing techniques have shown significant differences in gene expression patterns between resistant and susceptible phenotypes (Gaines *et al.* 2014; Pan *et al.* 2016). The difference in gene expression could be the result of modulating protein activity imposed by epigenetic mechanisms passed on through generations of resistant weed biotypes (Ho and Burggren 2010). For instance, epigenetic factors might change the post-translational modification of a protein in individuals experiencing a stress (Sadakierska-Chudy and Filip 2015). This modified protein could then alter the expression or activity of an enzyme that reduces the sensitivity of individuals to the stress (Guerra *et al.* 2015).

Therefore, exploring the role of epigenetic mechanisms on the pattern of gene expression at each generation during the early stages of evolution could be a very useful exercise in future investigations of the development of herbicide resistance in weed populations. We need to understand how epigenetic mechanisms modify the physiology of individual plants which are becoming resistant to herbicides, and to determine whether the epigenetic factors involved are transgenerational and irreversible.

### Fitness and evolutionary physiology

The reproductive success of plants is tightly linked to their fitness, and individuals with greater fitness have a higher frequency of offspring in the next generation (Vila-Aiub *et al.* 2015). Many studies have investigated the fitness of herbicide-resistant weed biotypes (Vila-Aiub *et al.* 2009). Plant scientists might look at the fitness of a herbicide-resistant phenotype relative to its susceptible counterpart in order to measure the fitness cost in the absence of environmental stress (Vila-Aiub *et al.* 2015). However, we are not aware of any studies into the link between physiological performance and the fitness of a phenotype at the early stage of evolution of herbicide resistance with an emphasis on understanding the adaptive systems that herbicide-resistant weedy plants evolve during the process of herbicide resistance selection. We also need to understand how phenotypic variation across generations affects the fitness of the plants under recurrent selection by herbicide applications. It would be particularly interesting to know how pre-existing phenotypic variation (before herbicide selection) within a population interacts with the physiological mechanisms that improve the fitness of herbicide-resistant weeds over the period of selection.

Alleles which control physiological functions in plants can influence the fitness of a phenotype (Orr 2003). The frequency of these alleles could change across generations due to the action of a selective agent (Orr 2009), resulting in phenotypes undergoing some physiological manifestation that diverts more resources into particular organs or functions which affect their fitness (Tian *et al.* 2003). The maximum number of resistance alleles that individuals within a population can accumulate depends on the number of resistance alleles that exist among the individual plants of that population, and how these resistance alleles affect the fitness of individuals (Délye 2013). However, there is a poor understanding of how the change in the frequency of resistance alleles over time during the selection process for herbicide resistance affects the fitness of individual plants. Many of the traits affecting the relative fitness of a phenotype are physiological responses of individuals, occurring as a result of the expression of specific genes at specific times of development, or under specific prevailing environmental conditions. Determining the links between genetic and non-genetics factors (e.g. phenotypic variations, epigenetic factors, the interaction of alleles, etc.) and physiological manifestations over the period of selection could provide further details about how the fitness of herbicide-resistant phenotypes is affected.

## Conclusions

Evolution of herbicide resistance by weed populations is a good example of how plant species can adapt to environmental constraints. Evolutionary biologists have investigated how weed populations evolve resistance to herbicides using artificial selection experiments. A number of new molecular research techniques have recently become widely available, enabling investigators to identify the relationship between variations in DNA sequences, transcriptomes, proteins, metabolite networks and physiological traits. These technologies can facilitate investigations into evolutionary physiology, allowing significant progress to be made in identifying and characterizing the functions of genes involved with herbicide resistance, and the interactions between corresponding proteins, as well as the regulators and pathways involved. Perspectives are outlined above that could lead to research being undertaken to help better understand non-target site mechanisms of herbicide resistance to herbicides, and thus identify factors that might be used to disrupt the evolution of resistance.

## Sources of Funding

Some funding was provided by Massey University for this work.

## Contributions by the Authors

H.G. conceived and developed the idea. H.G. and K.C.H. wrote the manuscript.

## Conflicts of Interest

None declared.
